# Research Progress on Emerging Viral Pathogens of Small Ruminants in China during the Last Decade

**DOI:** 10.3390/v14061288

**Published:** 2022-06-13

**Authors:** Li Mao, Wenliang Li, Fei Hao, Leilei Yang, Jizong Li, Min Sun, Wenwen Zhang, Maojun Liu, Xuenong Luo, Zilong Cheng

**Affiliations:** 1 Key Laboratory for Veterinary Bio-Product Engineering, Institute of Veterinary Medicine, Jiangsu Academy of Agricultural Sciences, Ministry of Agriculture and Rural Affairs, Nanjing 210014, China; mao-li@live.cn (L.M.); 20140010@jaas.ac.cn (F.H.); 20120034@jaas.ac.cn (L.Y.); 20150044@jaas.ac.cn (J.L.); 20170074@jaas.ac.cn (M.S.); 20110978@jaas.ac.cn (W.Z.); 20030022@jaas.ac.cn (M.L.); 20210005@jaas.ac.cn (Z.C.); 2 Jiangsu Key Laboratory for Food Quality and Safety-State Key Laboratory Cultivation Base, Ministry of Science and Technology, Nanjing 210014, China; 3 State Key Laboratory of Veterinary Etiological Biology, Lanzhou Veterinary Research Institute, Chinese Academy of Agricultural Sciences, Lanzhou 730046, China; luoxuenong@caas.cn

**Keywords:** small ruminants, PPRV, CPIV3, BDV, ENTV, CpHV-1, enterovirus

## Abstract

China is the country with the largest number of domestic small ruminants in the world. Recently, the intensive and large-scale sheep/goat raising industry has developed rapidly, especially in nonpastoral regions. Frequent trading, allocation, and transportation result in the introduction and prevalence of new pathogens. Several new viral pathogens (peste des petits ruminants virus, caprine parainfluenza virus type 3, border disease virus, enzootic nasal tumor virus, caprine herpesvirus 1, enterovirus) have been circulating and identified in China, which has attracted extensive attention from both farmers and researchers. During the last decade, studies examining the etiology, epidemiology, pathogenesis, diagnostic methods, and vaccines for these emerging viruses have been conducted. In this review, we focus on the latest findings and research progress related to these newly identified viral pathogens in China, discuss the current situation and problems, and propose research directions and prevention strategies for different diseases in the future. Our aim is to provide comprehensive and valuable information for the prevention and control of these emerging viruses and highlight the importance of surveillance of emerging or re-emerging viruses.

## 1. Introduction

China is the country with the largest number of sheep and goats in the world, with an average stock of >300 million. In recent years, the traditional grazing pattern in pastoral areas and the backyard feeding pattern in nonpastoral areas have gradually decreased, while the intensive and large-scale sheep/goat raising industry has developed rapidly. With the development of the industry, frequent trading, allocation, and transportation, the introduction and prevalence of new diseases have imposed serious harm. In particular, during 2013–2014, the introduced peste des petits ruminants (PPR) spread to nearly all of the provinces of China in a short period of time, resulting in substantial economic losses [1]. Veterinarians and researchers mainly focused on the diseases and several emerging viral pathogens present in China [2,3,4,5,6,7]. Researchers have conducted studies investigating aspects of etiology, epidemiology, pathogenesis, diagnostic methods, and vaccine development, which promote the understanding, prevention, and control of related pathogens. In this review, we focus on and discuss the latest findings and research progress related to these newly identified viral pathogens in China.

## 2. Peste des Petits Ruminants Virus (PPRV)

### 2.1. Etiology and Epidemiology

PPRV is classified into the genus *Morbillivirus* in the family *Paramyxoviridae* and represents an acute and highly contagious viral pathogen of small ruminants, mainly infecting goats and sheep [8]. Since its first identification in early 1940s in Cote d’Ivoire, PPRV has steadily expanded its geographical distribution to approximately 70 countries in Africa, the Middle East, and parts of Asia over the last 20 years [9]. In the worst situations, PPRV-infected animals may die within 10–12 days after the onset of pyrexia, and the main clinical manifestations include fever, tears, snot, stomatitis, pneumonia, and diarrhea. The related morbidity rate is 100%, with up to 90% mortality. In areas where the disease is endemic, the mortality rate may be lower; however, the disease exerts an insidious impact, hampering the development of lambs and kids and compromising the immune defense of adult animals against other bacterial diseases. As one of the most damaging diseases of small ruminants, PPR causes USD 1.5 to 3 billion in losses each year in affected regions, impacting more than 330 million of the poorest people worldwide, many of whom depend on these ruminants for their livelihoods [9].

According to the timelines, the prevalence of PPR in China is divided into three stages: 2007–2013, 2013–2014, and after 2014. The first two stages have been discussed in a previous review [1]. PPRV infection in China was first reported in the border region, Ngari of western Tibet, in July 2007 [10]. In subsequent years, the epidemic spread slowly eastward through mixed herd grazing and broke out in the Nagqu and Ngari regions of Tibet in 2008 and 2010, respectively. The outbreak was successfully eliminated by the implementation of powerful control measures [11].

At the end of 2013, PPR re-emerged in China, and then its spillovers successively occurred in 22 provinces, autonomous regions, and municipalities in 2014. The first case was reported in Xinjiang in November 2013 [12]. Soon afterward, the wave of outbreaks rapidly spread from west to the east into 21 other provinces, forming a pandemic animal disease. The phylogenetic analysis based on the N gene fragment indicated that the circulating strains shared a close relationship with the epidemic strains detected in Tajikistan and Pakistan in 2012, the countries bordering Xinjiang [11,13,14].

After 2014, compulsory immunization with the attenuated Nigeria 75/1 strain was conducted. The PPR pandemic was well controlled and became scattered throughout China. To date, the Ministry of Agriculture and Rural Affairs of China has reported PPR cases (http://www.moa.gov.cn/govpublic/, accessed on 10 May 2022) in Xinjiang (2016, 2020, 2021, and 2022), Shanxi (2016), Ningxia (2016), Tibet (2021), Guizhou (2016), Jiangsu (2018), Hunan (2018), Heilongjiang (2018), Qinghai (2018, 2020, and 2021), and Liaoning (2020).

In addition to being prevalent in domestic animals, PPRV has also been repeatedly reported in wild animals. In 2008, PPRV infection was identified in the wild host bharal in Ge’gyai County of Tibet, and the fusion protein (F) and N gene segments were closely related to other circulating PPRV strains recently identified in sheep and goats from Tibet [11]. Subsequently, the prevalence of PPRV in other wildlife hosts was well characterized. Four wild species, Capra ibex (*Capra ibex sibirica*), argali (*Ovis ammon*), wild ibexes, and Goitered gazelle (*Gazella subgutturosa*) from Xinjiang, were detected as positive, and five wildlife-origin isolates from the aforementioned samples were identified as lineage Ⅳ [12,15,16]. PPRV infection was also observed in wild Przewalski’s gazelle in Gansu and wild rock sheep in Qinghai Province [17,18]. Wild species may serve as sentinels indicating the spillover of PPR from domestic animals; on the other hand, these PPR cases provided evidence that the risk of PPRV was maintained due to the presence of natural foci. According to the epidemiological data, the risk analysis model was established. A spatiotemporal cluster analysis was performed to determine whether areas and time periods with significant aggregation of PPR outbreaks occurred. The most likely disease cluster was mainly located in southeastern China, and the area included Henan, Anhui, Jiangsu, Hubei, Zhejiang, and Jiangxi provinces. The second class cluster was located in southern China and included Chongqing, Hunan, Guizhou, Yunnan, and Guangzhou. The third class cluster was located in northeastern China, including Heilongjiang and Jilin provinces [19].

PPRV has continued to expand its geographic boundaries, reaching regions previously not infected and putting hundreds of millions of both domestic small ruminants and wildlife at risk of infection. However, the occurrence of PPRV infection in previously uninfected regions, together with the mixing of lineages in endemically infected countries, highlights the dynamic and transboundary nature of this disease. The expansion of the PPR epidemic has been known for many decades, but considerable time was needed to increase international interest and to bring PPR to the status of a priority disease for livelihood and food security.

### 2.2. Virus–Host Interaction

Viruses have been reported to interact strongly with specific host pathways that are usually involved in the control of infections. Based on accumulating evidence, interferon (IFN) and autophagy-related pathways are the major targets of PPRV during the course of viral infection. Upon viral invasion and replication, IFN and autophagy are induced as an innate immune mechanism to control infection. Studies have suggested that IFN and autophagy not only serve as protective functions for cell survival but also play a role in cellular defenses against PPRV infection.

#### 2.2.1. Interaction of PPRV and IFN-Related Pathways

Previous studies have documented crucial roles for viral proteins in blocking type I and type II IFN responses, and various mechanisms have been proposed for these effects. Upon PPRV infection, mitochondrial antiviral-signaling protein (MAVS, also known as VISA, IPS-1, or CARDIF) plays an essential role in the activation of the type I IFN pathway [20]. PPRV infection significantly induces ATG13 (autophagy-related 13) expression, and ATG13 exerts an obvious antiviral effect on PPRV infection in vitro [21]. Proteomic analysis showed that the E3 ubiquitin-protein ligase FANCL inhibited PPRV infection by enhancing type I IFN and IFN-stimulated gene expression through an increase in TBK1 phosphorylation [22].

Both N and phosphoprotein (P) proteins were demonstrated as novel IFN response antagonists. The P protein interacted with STAT1 and subsequently inhibited STAT1 phosphorylation, whereas the N protein neither interacted with STAT1 nor inhibited STAT1 phosphorylation or dimerization, suggesting that the antagonistic effects of the N and P proteins are different [23]. Moreover, the N protein participated in suppressing interferon regulatory factor 3 (IRF3) function and type I IFN production by interfering with the formation of the TBK1–IRF3 complex [24]. The P protein significantly suppressed RIG-I-like receptor pathway signaling and impaired the expression of IFN-β and ISGs by targeting IRF3 in both human embryonic kidney 293T cells and primary goat fibroblasts [25]. In addition to the N and P proteins, the C protein significantly inhibits the phosphorylation of STAT1, interferes with signal transduction through the JAK–STAT signaling pathway, and exerts antagonistic effects on the expression of IFN-β through both MAVS- and RIG-I-mediated pathways [26]. Notably, miRNAs were also identified to be involved in the PPRV–IFN response interaction. PPRV V protein alone was sufficient to induce miR-3 expression, and miR-3 inhibits type I IFN signaling, thus promoting infection [27] (Table 1).

#### 2.2.2. PPRV Infection and Autophagy

Despite the key role of the IFN pathway in pathogen control, autophagy plays an important role in PPRV pathogenesis. PPRV infection-triggered autophagy was first reported in 2013 in Vero cells [28]. Immediately upon invasion, PPRV induced a first transient wave of autophagy via a mechanism involving the cellular pathogen receptor NECTIN4 and an AKT–MTOR (mechanistic target of rapamycin kinase)-dependent pathway. Then, the binding of viral protein H to NECTIN4 ultimately induced another wave of autophagy and inactivated the AKT–MTOR pathway, which is a critical step in controlling the infection. Moreover, the expression of immunity-related GTPase M (IRGM) and heat shock protein family A (Hsp70) member 1A (HSPA1A) was significantly upregulated upon PPRV infection. The interaction of IRGM with PPRV-C and HSPA1A with PPRV-N was sufficient to induce autophagy through the IRGM-HSPA1A pathway [29]. PPRV infection triggered a complete autophagy response that was most likely mediated by the C and N proteins. Virus-induced autophagy inhibits the caspase-dependent apoptosis pathway and thus promotes viral replication and maturity in host cells [30]. The role of autophagy in the secretion of infectious PPRV was also studied. ATG7 (autophagy-related 7)-mediated autophagy enhances exosome secretion and exosome-mediated PPRV transmission. TSG101 may be involved in the sorting of infectious PPRV RNA genomes into exosomes to facilitate the release of PPRV through the exosomal pathway. Inhibition of PPRV-induced autophagy or TSG101 expression might be used as a strategy to block exosome-mediated virus transmission [31].

#### 2.2.3. Additional Virus–Host Interactions

Cyclophilin A (CypA) has been reported to inhibit or facilitate viral replication, and evidence has revealed that CypA suppresses PPRV replication through its PPIase activity, while the H protein is responsible for the reduction in CypA, which depends on the lysosome pathway [32]. Another important part of the innate immune response was proven to participate in the activation of the NF-κB signaling pathway and NLPR3 inflammasome upon exposure to PPRV. PPRV promoted the secretion of interleukin 1β (IL-1β) by activating the NF-κB signaling pathway and the NLRP3 inflammasome. Moreover, PPRV replication and protein synthesis are essential for NLRP3 inflammasome activation [33]. PPRV induced tumor necrosis factor-like weak inducer of apoptosis (TWEAK) expression by suppressing the expression of miR-1, a novel miRNA directly regulating the TWEAK gene negatively. Moreover, viral replication is required for the inhibition of miR-1 expression during PPRV infection, and the nonstructural V protein of PPRV plays an important role in miR-1-mediated TWEAK upregulation [34].

### 2.3. Diagnosis

Diagnosis remains a cornerstone for the global control of PPR and eradication strategies. Since the mid-1980s, the diagnosis of PPR has constantly been improved, benefiting from advances in biotechnology, bioinformatics, and miniaturization of electronic devices. Tools are now available for the rapid and specific diagnosis of PPR by diagnosticians with different skill levels using the equipment available in the test laboratory. Based on the World Organization for Animal Health (OIE)-recommended methods [9] and reported detection methods, a series of diagnostic tools have been developed for detecting PPR by many groups in China since its first outbreak in Tibet.

#### 2.3.1. Etiological Detection Methods

OIE recommended pen-side tests for PPR diagnosis by specialized and nonspecialized diagnosticians, including serum-based tests (ELISA) for the detection of viral antigens, viral nucleic acid detection (reverse transcription polymerase chain reaction (RT–PCR) and real-time quantitative RT–PCR (RT–qPCR)), and virus isolation. PRRV isolation using primary cells (bovine, ovine, and caprine kidney and lung epithelial cells) requires multiple, sequential blind passages and up to weeks in culture before the development of any cytopathic effect [35]. This method requires professional skills and is time-consuming and difficult to achieve in poorly equipped laboratories. In China, RT–PCR and RT–qPCR for the viral N gene are the molecular detection methods adopted in China’s national standard “Diagnostic techniques for Peste des petits ruminants (GB/T 27982-2011)”. In addition, lineage Ⅳ-specific RT–qPCR [36] and loop-mediated isothermal amplification assay (RT-LAMP) [37] were established. Recently, reverse transcription recombinase polymerase amplification (RT–RPA) assays using real-time fluorescent detection (real-time RT–RPA assay) and lateral-flow strip detection (LFS RT–RPA assay) were reported to have high specificity and detection sensitivity [38,39,40]. The chip digital PCR (cdPCR) assay was also developed; cdPCR showed good stability and specificity, and its sensitivity was 1000 times higher than that of RT–PCR [41].

The antigen-captured ELISA was also developed. An indirect ELISA based on the recombinant goat signaling lymphocyte activation molecule (SLAM) as the capture ligand was successfully developed for the detection of the PPRV antigen. The assay was highly specific for PPRV with no cross-reactions to other viruses [42]. In addition, a simplex suspension array was also established, with a detection limit of 5.78 × 10^4^ copies/mL, and it was shown to be 100% consistent with the conventional RT-PCR [43].

#### 2.3.2. Serological Detection Methods

Serological methods, such as ELISA, which is recommended by OIE, have been used for PPR diagnosis in a virus-free region. N protein-based ELISA has been reported worldwide, and the authors and Wang et al. have developed blocking ELISA methods, which share a high coincidence rate with commercial kits [1]. In the study by Sun et al., the recombinant N proteins were successfully expressed in two different prokaryotic vectors (pET-30a and pET-32a). The double-antigen S-ELISA antibody detection method based on the combination of N antigens presented a better detection effect than a commercial PPRV kit [44]. Epitope-based ELISA is another choice; four synthesized epitope peptides based on the B cell epitopes of H protein with excellent reactivity were coated as antigens, and an iELISA test method was developed for detecting PPRV antibodies [45]. In addition, epitope peptides of Tibetan PPRV protein were used to prepare hyperimmune antisera and develop a cELISA for detecting antibodies [46]. Recently, several alternative antibody detection methods have also been developed. A fast and ultrasensitive test-strip system combining quantum dots (QDs) with a lateral-flow immunoassay strip (LFIAS) was developed for PPRV antibody detection [47]. A competitive chemiluminescent assay kit for PPRV antibodies was established by combining the obtained PPRV N fusion protein with relevant monoclonal antibodies. This method was capable of quantitatively detecting the antibody against PPRV in sheep serum, with high sensitivity and specificity [48].

### 2.4. PPRV Vaccines

#### 2.4.1. Live Attenuated Vaccines

One of the key conditions for the success of the global PPR eradication program was the use of a highly efficacious vaccine in protecting animals against all PPRV strains. Current PPRV attenuated Nigeria 75/1 and India/Sungri/96 strain vaccines provide complete clinical protection against challenge with all four lineages of PPRV [49]. However, both vaccines are thermolabile, and they require uninterrupted maintenance of the cold chain until their application to the animal to avoid their thermal inactivation.

Emergency vaccination with the Nigeria 75/1 vaccine protected the animals in the first outbreak in China. The vaccine has been widely used in China since the second wave of PPR outbreaks during 2013–2014. The currently commercially available vaccines in China (Nigeria 75/1 and Clone 9 strain originating from Nigeria 75/1) are in freeze-dried form and are stable for at least two years at 2 °C to 8 °C [9]. Once the vaccine is reconstituted, it must be utilized as soon as possible (not later than 30 min after dilution). Compulsory immunization is still performed in many provinces of China. Upon clinical application, the presence of maternal antibodies should be considered to achieve the best vaccination effect. Based on our research, the duration of maternal antibodies on different farms varied substantially, with most responses lasting from 1 to 3 months, and maternal antibodies seriously interfered with the PPRV vaccine [50]. Therefore, a rational vaccination procedure is needed for different farms to avoid the interference of maternal antibodies. Although the vaccine can confer strong protective immunity in sheep and goats, it is unable to differentiate infected from vaccinated animals (DIVA).

#### 2.4.2. Vector Vaccines

For a long time, the generation of a humoral immune response by an attenuated vaccine was considered sufficient and the most important correlate of vaccination with protection. However, new generation PPR vaccine candidates have been developed worldwide in the past several years, which will probably serve as alternatives to conventional vaccines in the future. These candidates, especially viral vector vaccines, have been developed in China and have been proven to have the ability to induce humoral protection in vivo. Goat poxvirus-vectored PPR vaccine, a promising candidate DIVA vaccine, was developed. High levels of long-lasting neutralizing antibodies were induced in more than 80% of vaccinated goats and sheep over 6 months and protected animals from virulent PPRV challenge [51]. Recently, recombinant PPRV was studied using reverse genetic techniques, which might provide a useful tool for investigating biology and pathology and developing new vaccines. Recombinant PPRV stably expressing GFP was constructed and replicated as well as the parental virus, allowing a more rapid and higher throughput assessment of viral neutralization antibody via the virus neutralization test (VNT), as well as the development of new vaccines [52]. Furthermore, a recombinant PPRV expressing the foot-and-mouth disease virus (FMDV) VP1 gene (rPPRV/VP1) was generated without impairing immunogenicity by inducing the production of a neutralizing antibody against PPR in goats [53]. In addition, a suicidal DNA vaccine based on the Semliki Forest virus (SFV) replicon expressing the PPRV H gene was constructed. It induced the production of neutralizing antibodies and lymphocyte proliferation responses in mice, suggesting that it might represent a promising new approach for vaccine development against PPR [54].

#### 2.4.3. Virus-like Particle Vaccines

Currently, vaccination with virus-like particles (VLPs) presents considerable promise as a prophylactic approach and has been employed in a variety of viral vaccine designs. The glycoproteins H and F of PPRV are considered important for inducing protective immune responses; therefore, these two proteins are more commonly expressed as immunogens in VLPs. VLPs of PPRV generated in a baculovirus system expressing PPRV matrix (M) protein and H or F protein showed similar morphology to the native virus. Vaccination with these VLPs (PPRV-H, PPRV-F) in mice and goats elicited the production of specific neutralizing antibodies and promoted lymphocyte proliferation. The immune responses induced by the PPRV-H VLPs without adjuvants were comparable to those induced by the PPRV vaccine [55]. Another PPRV VLP-based vaccine was constructed by expressing the M, H, F, and N proteins in insect cells, inducing a robust IFN-γ response and stimulating an immune response in inguinal lymph nodes. PPRV VLPs induce the production of neutralizing antibodies at the same magnitude as the PPRV Nigeria 75/1 vaccine and elicit a cellular immune response [56]. A similar recombinant baculovirus was constructed to coexpress the PPRV M, H, and N proteins in insect cells, inducing the production of both types of antibodies in mice. Additionally, compared to the lineage Ⅱ Nigeria 75/1 vaccine strain VLPs, the VLPs derived from the virulent lineage Ⅳ Tibet/30 strain are more immunogenic, inducing more potent and robust humoral and cell-mediated immune responses in vaccinated animals [57]. The aforementioned studies suggest the potential prospects of VLP-based vaccines against PPR [58].

### 2.5. Prevention and Control of PPR

The Food and Agriculture Organization of the United Nations (FAO) and OIE adopted the PPR Global Control and Eradication Strategy (PPR-GCES) in 2015 [9]. The PPR-GCES is based on the strong scientific, economic, and social case for PPR control and eradication combined with the successful experience with rinderpest virus (RPV) eradication [59]. In China, the National Medium- and Long-Term Program for Animal Disease Prevention and Control (2012–2020) has been implemented, and PPR is listed for disease eradication [1].

The first step in completing the global eradication plan is to further study the mechanism of PPRV transmission in wild animals and prevent the formation of natural foci. Second, a stable, accurate, cheap, and easily operated PPRV detection method must be developed that can be used in undeveloped regions or the field environment. Countries and regions that have already controlled and eliminated PPR should strengthen the prevention and control of imported PPR, and others that are still endemic should promote vaccination and rapid detection technology.

In China, imported PPR has still occurred sporadically in different regions in recent years [60]. The occurrence of imported PPR cases is closely related to the continuous epidemic of PPR around China, especially in southwest neighboring countries. Due to the serious PPR epidemic situation in neighboring countries, the current prevention and elimination measures of PPR in China should focus on preventing the importation of infected animals from abroad in addition to continuing the immunization of domestic sheep/goats. At present, the main methods of importing affected animals are transboundary contact between PPRV-infected wild animals and domestic animals and the import of infected domestic animals. In addition to strengthening herd management and wildlife migration monitoring in border regions, quarantine organizations such as customs should focus on PPRV detection. The development of more convenient and efficient PPRV detection methods for customs, quarantine stations, and animal farms is very important. Fortunately, a variety of increasingly mature new detection techniques are used in PPRV detection. Vaccination is the key to controlling and eradicating PPR in endemic countries. The currently available vaccine provides effective protection against PPR. A new generation of PPR vaccines is also being developed that will provide an effective approach for differentiating immunized animals from infected animals and ultimately clearing PPR.

## 3. Caprine Parainfluenza Virus Type 3 (CPIV3)

### 3.1. Etiology and Pathogenesis

**Figure 1:**
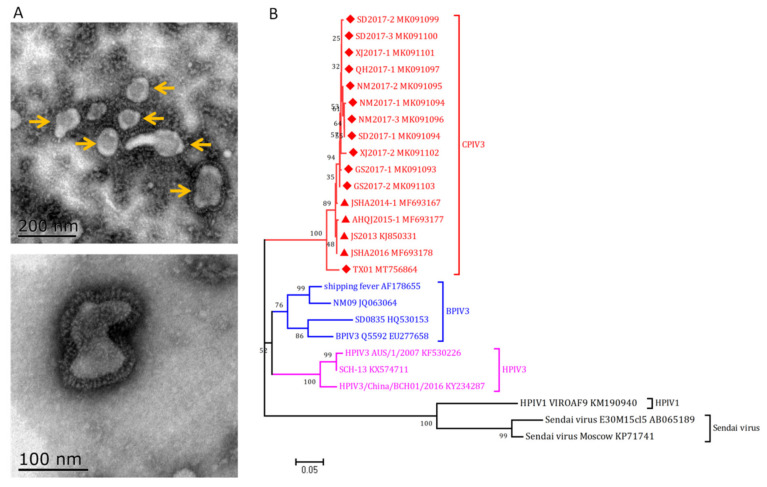
Morphology and phylogenetic analysis of CPIV3. (**A**) Morphology observation of CPIV3 under electron microscope. Two hundred milliliters of virus stock was centrifuged at 8000× *g* for 0.5 h to remove the cell debris; the supernatant was then ultracentrifuged at 100,000× *g* for 2 h. The resulting pellet was dissolved in 1mL PBS and stained with phosphotungstic acid (PTA), blotted dry, and examined with an electron microscope (H-7650, HITACHI). Spherical to pleomorphic virions with a diameter of 100–200 nm were observed. (**B**) Phylogenetic analysis of the reported CPIV3 strains and reference respiratory strains based on M gene sequences using MEGA 7 software. The red triangle and rhombus indicate the goat and sheep originated strains, respectively.

CPIV3, a novel member of *Paramyxoviridae* family, was first identified in goats with respiratory diseases in China [2]. Based on the genome sequence and phylogenetic analysis, CPIV3 belongs to the genus *Respirovirus* together with human parainfluenza virus types 1 and 3 (HPIV1 and HPIV3, respectively), bovine parainfluenza virus type 3 (BPIV3), and Sendai virus (SeV). Similar to other *Respirovirus* members, CPIV3 is an enveloped virus with a non-segmented negative-stranded RNA genome that encodes six structural proteins, namely nucleoprotein (N), phosphoprotein (P), matrix protein (M), fusion protein (F), hemagglutinin-neuraminidase protein (HN), and large protein (L) [61]. CPIV3 has a spherical to pleomorphic shape with a diameter of 100–200 nm (Figure 1A, yellow arrow). The virions consist of a nucleocapsid surrounded by a lipid envelope. Viral transmembrane glycoproteins HN and F mediate attachment and entry of the virions into host cells and delivery of the nucleocapsid into the target cells [62].

To date, the antigenic and genetic variation among CPIV3 isolates is at a low level. Phylogenetic analysis demonstrated that these strains belonged to the same cluster (Figure 1B), although a novel lineage of CPIV3 (TX01 strain) was proposed from sheep herds that suffered severe respiratory disease [63]. An ovine PIV3 recently identified in northeast China was distinct from the reported PIV3 strains and might be another novel species of the genus *Respirovirus* [64], but the genome sequence is not available; more epidemiology and etiology studies are needed to confirm this conclusion.

Similar to BPIV3, single CPIV3 infection usually induces sub-clinical or mild symptoms. However, viral infection usually causes immunosuppression and severe bronchopneumonia from secondary bacterial infections [62]. Thus, in instances of stress such as transportation, weather change, and co-infection with other bacterial pathogens (*Mannheimia haemolytica*, *Pasteurella multocida*, *Mycoplasma ovipneumoniae*, etc.), CPIV3 infection causes severe symptoms and results in high mortality in field conditions [61,65,66,67]. The results from experimental infection provided evidences for the pathogenicity of CPIV3 [2,68]. Upon CPIV3 infection, clinical signs such as cough, nasal discharge, and fever can be observed. Pathological changes are mainly found in the lungs and tracheas. Mild to moderate diffuse purple consolidation is the major gross lesion observed in the lungs. Under histopathological examination, thickened alveolar walls, decreased alveolar space, and increased amounts of inflammatory cell infiltration could be observed in lungs; the tracheas of infected goats showed shortened or disappeared cilia and submucosal edema. Viremia and virus shedding from nasal discharge and feces were identified from 3 to 21 days post-infection (dpi). Successful airborne horizontal transmission of CPIV3 was also identified [68]. Furthermore, in the study of Ma et al., the CPIV3-infected sheep displayed syndromes such as depression, cough, and fever. Virus shedding, viremia, and pathological changes in the lungs were similar to those in goats [63]. In conclusion, it is proposed that CPIV3 is one of the important components of respiratory disease for goats/sheep.

### 3.2. Epidemiology

**Figure 2:**
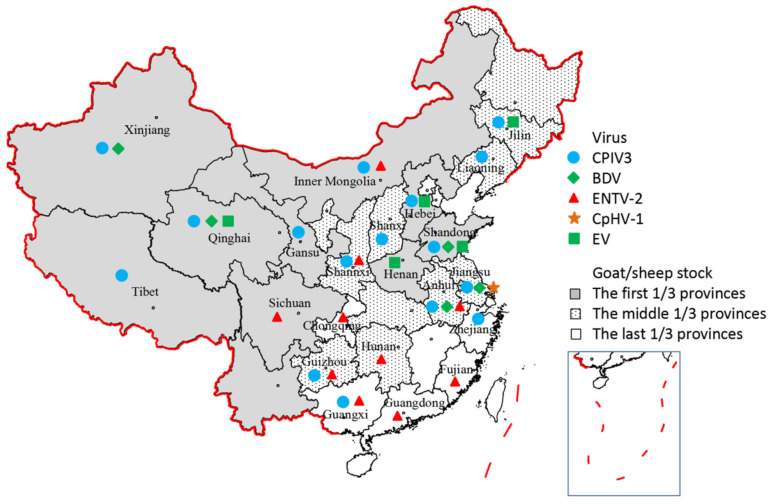
Prevalence of the emerging viruses in different provinces of China. Different symbols represent different emerging viruses. The different color in each province region represents the ranking of goat/sheep stock.

After the identification of CPIV3 in 2014, epidemiology studies were continually performed by our team and several other researchers. Based on the serological test results, CPIV3 infection was confirmed in 16 of the 17 selected provinces in China (Figure 2), and a high level of viral antibody in goat and/or sheep herds was identified (Table 2) [65,66,69,70]. The seropositive rates in goats and sheep ranged were 0–41.94% and 10.96–84.76%, respectively. The sheep in northern and northwestern provinces, such as Inner Mongolia, Xinjiang, Qinghai, and Gansu, showed extremely high levels of CPIV3 antibody (61.86–84.76%, Table 2). Bi et al. analyzed serum samples from six different cities in Guangxi, and 26.97% (171/634) were CPIV3-positive [69]. Chen et al. reported a positive rate of 14.50% (108/745) for goat serum samples in Shandong using a recombinant N protein-based indirect ELISA [70].

To date, 16 CPIV3 strains (12 of sheep origin and 4 of goat origin, Figure 1B) have been identified in clinical samples, and these viruses share high homology with each other (partial M gene sequences, 95.06–99.74% homology). Furthermore, six full-length genome nucleic acid sequences were obtained and submitted to GenBank (MK091103, KT215610, MF693178, MF693177, MF683167, MT756864), and they possessed 97.58-99.91% homology as determined by BLAST analysis.

### 3.3. Virus–Host Interaction

The virus propagates in and induces cytopathic effects (CPEs) on several cell lines (Madin–Darby bovine kidney (MDBK), Vero, and HEK-293T) and primary caprine/ovine cell cultures (kidney cells, testis cells, and bronchial/tracheal epithelial cells). Based on our experience, MDBK cells are the first choice for virus isolation and proliferation. Effective replication of CPIV3 requires cholesterol in both the cell membrane and viral envelope, especially in the viral envelope. Cholesterol-rich lipid rafts are critical for the entry and infection of CPIV3 in both MDBK cells and goat bronchial epithelial cells [71].

Upon viral infection, host cells produce a series of immune responses to restrict virus proliferation. RNA-Seq analysis of CPIV3-infected MDBK cells revealed many differentially expressed genes (DEGs) involved in immune system processes, metabolic processes, and signal transduction. Further analysis revealed that seven interferon-stimulated genes (ISGs) were upregulated (IFI6, ISG15, OAS1Y, OAS1Z, Mx1, Mx2, and RSAD2), and these ISGs were confirmed to inhibit CPIV3 infection in MDBK cells [72].

In contrast, CPIV3 also employs different strategies for immune escape and efficient replication in target cells: (1) Inducing exosome secretion and modulating autophagy: CPIV3 infection promotes the secretion of large amounts of exosomes that are loaded with viral proteins and RNA. These exosomes transfer viral components to recipient cells and establish a productive infection, which contributes to viral replication. In addition, autophagy is inhibited by CPIV3 exosomes, and exosome-packaged miR-126-3p_2 was identified as an important regulatory factor during this process. Inhibition of autophagy is one of the factors promoting viral replication through exosome-mediated CPIV3 infection [73]. (2) Inducing apoptosis: CPIV3 infection induces apoptosis in both MDBK cells and goat tracheal epithelial cells by activating both the intrinsic and extrinsic pathways, which subsequently increases CPIV3 replication. Mechanistically, the ability of CPIV3 to induce apoptosis is mediated by the N protein [74,75]. One of the host cellular miRNAs, bta-miR-98, which markedly reduces CPIV3 replication by regulating apoptosis, is downregulated during CPIV3 infection and promotes viral replication. (3) Modulating Type I IFN (IFN-I) signaling: CPIV3 infection reduces cellular STAT1 expression and phosphorylation to circumvent the host IFN-I response. Viral accessory proteins C and V were identified to antagonize the IFN-α antiviral effect, and protein C inhibited STAT1 signaling by reducing the IFN-α-induced increase in the level of phosphorylated STAT1 (pSTAT1). A highly variable region (VR2) is involved in this process [76]. Furthermore, viral infection significantly downregulated the expression of bta-miR-222, which was identified to increase IFN-I expression and suppress CPIV3 replication by directly targeting interferon regulatory factor 2 (IRF2) [77].

### 3.4. Diagnosis

The diagnosis of CPIV3 infection could be performed by: (1) detection of virus RNA in respiratory samples by RT-PCR or RT-qPCR; (2) detection of viral-specific antibodies; (3) detection of viral antigen; and/or (4) virus isolation. Viral-specific antibody detection is usually used for serological investigation, while virus isolation and viral nucleotide acid and antigen detection are always used for agent examination and disease diagnosis. Active infection can be diagnosed by virus isolation or molecular tests from nasal swabs and respiratory tissues [78]. Due to the short period of virus shedding, the sampling time is critical for pathogen detection, to obtain a definitive diagnostic result.

#### 3.4.1. Virus Isolation and Identification

Virus isolation is the “gold standard” method for CPIV3 detection in vitro. It can be performed in MDBK cells or primary caprine/ovine cells. Fresh nasal swab, lung, and bronchial/tracheal samples are frequently used for virus isolation. The presence of CPIV3 in the cell cultures is further identified using hemagglutination (HA) tests, indirect immunofluorescence assays (IFAs), and molecular tests. However, virus isolation is labor-intensive, time-consuming, and not suitable for large-scale sample screening. In addition, based on viral-specific monoclonal antibodies (mAbs), a double mAb sandwich ELISA (unpublished) has been developed for CPIV3 antigen detection, which will facilitate the detection process, especially for large-scale pathogenic screening.

#### 3.4.2. Molecular Tests

Viral nucleotide acid detection is widely used in clinical diagnosis. A pair of primers reported for BPIV3 has been used for CPIV3 detection (Table 3) [79]. Furthermore, following the sequencing and alignment of CPIV3 genomes with those of BPIV3, RT–PCR and TaqMan real-time RT-qPCR assays have been developed in our lab for the specific detection of CPIV3, which have no cross-reactivity with BPIV3 (Table 3) [67,80]. These methods have been used in epidemiological and pathogenic studies.

#### 3.4.3. Serological Tests

Serological tests are widely used to determine the prevalence of CPIV3 infection in a flock, region, or country. Antibody-positive results usually imply the introduction and prevalence of CPIV3. Virus neutralization test (VNT) and hemagglutination inhibition (HI) assays (using guinea pig erythrocytes) are commonly used. VNT is labor-intensive and time-consuming; the HI assay is also relatively labor-intensive. Therefore, these two methods are not suitable for the detection of large numbers of samples.

ELISA is a powerful high-throughput screening tool that is suitable for large-scale epidemiological investigations. Two indirect ELISAs using prokaryotically expressed truncated N protein as a coating antigen were developed for viral-specific IgG antibody detection [81]. In addition, a mAb-based blocking ELISA was developed for the detection of viral IgG and IgM antibodies and used in our seroepidemiological studies, the results revealed a good qualitative correlation with VNT and HI results [70].

### 3.5. Prevention and Control

The prevention and treatment of CPIV3-associated diseases are similar to the methods used for BPIV3 [62]. Vaccination is the most effective method, but a commercially available vaccine is still unavailable worldwide. General measures, such as ensuring optimized environmental conditions, a sufficient supply of nutrients, and a reduction in stress factors, should be applied to minimize the development of clinical disease in CPIV3-positive farms. In addition, biosecurity strategies, including quarantine, cleaning and hygiene procedures, disinfection, and “all-in all-out” procedures, are also powerful approaches for CPIV3 prevention, both in CPIV3-positive and CPIV3-free farms.

Treatment of CPIV3-infected animals mainly relies on supportive measures to maintain proper energy and electrolyte balance. Symptomatic treatment should be administered if the diseased animals present with fever, nasal discharge, cough, asthma, and other symptoms. Antitussive/antiasthmatic drugs such as aminophylline and NH_4_Cl could be used; in clinical practice, treatment with traditional Chinese medicine compounds usually achieves good results. For anti-inflammatory treatment, nonsteroidal anti-inflammatory drugs are the first choice to reduce fever and the damaging inflammatory response in the lungs without side effects like those of glucocorticoids. If secondary bacterial infections have been confirmed, susceptible antimicrobials should be chosen as the first prescription.

## 4. Border Disease Virus (BDV)

### 4.1. Etiology and Genotyping

Border disease (BD) is a major viral disease of sheep and goats caused by BDV. The disease was first recognized in the border region of England and Wales [82]. The clinical signs of infected sheep herds mainly include barren ewes, abortions, stillbirths, and the birth of small weak lambs [83]. Acute mucosal disease cases have occasionally occurred in the new BDV outbreaks. Vertical transmission plays an important role in the epidemiology of the disease. Infection of fetuses can result in the birth of persistent infection (PI) lambs. The PI animals are the most potent source of infection and play an important role in BDV spread [84].

BDV is an enveloped, positive-sense single-stranded RNA virus that belongs to the genus *Pestivirus* within the family *Flaviviridae*. Recently, a revision of the taxonomy of the genus *Pestivirus* was proposed, naming *Pestivirus* A to K [85,86]. *Pestivirus* H (atypical ruminant *Pestivirus*, or HoBi-like viruses) was identified in various bovine species, *Pestivirus* E (Pronghorn antelope *Pestivirus*) was reported in antelope, and *Pestivirus* I (Aydin like *Pestivirus*) was identified in small ruminants [86]. The *Pestivirus* infections in small ruminants in China mainly include BDV and bovine viral diarrhea virus (BVDV). The earliest BVDV infection in a Chinese sheep herd was reported in the 1990s, while BDV was first detected in a goat herd suffering diarrhea in Anhui Province in 2012 [87].

Based on genetic alignment of the 5′-UTR and/or N^pro^ region, BDV field isolates from sheep and goats are phylogenetically divided into at least eight genotypes (BDV-1 to BDV-8) [86,87,88]. Other sheep-derived pestiviruses, such as Tunisian, Tunisian-like, Aydin-like (*Pestivirus* I, Turkey), and a new emerging ovine *Pestivirus* (OVPV), were identified as responsible for BD-like syndromes [89,90]. However, these viruses were phylogenetically divergent from current BDV and showed a closer relationship with classical swine fever virus (CSFV) than BDV [86]. The BDV isolates identified from goats and sheep in China belong to the BDV-3 group. We refer the reader to the review by Righi et al. that describes the spread of different BDV genotypes and genetic heterogeneity of BDV worldwide in different host species [86].

### 4.2. Epidemiology and Cross-Species Transmission

Generally, BDV transmission relies on direct contact with infected animals via the oronasal route. Vertical transmission via the placenta plays an important role in the epidemiology of BD. The occurrence of BDV infection in domestic and wild animals, mostly in sheep, has been confirmed in different countries worldwide [8].

BDV was first identified in China in goats suffering severe and unremitting diarrhea in 2012. Three different BDV strains were identified as BDV-3 using RT–PCR, sequencing, and electron microscopy [4]. Afterward, one sheep infected with the original BDV strain was reported from a slowly grown PI lamb, named JSLS12-01. Experimental infection of sheep with JSLS12-01 produced clinical signs of depression and short-period (5 days) mild fever [3]. In 2013, sera and tissue samples were collected from diseased and healthy goat/sheep herds in Jiangsu Province. Twenty-seven of ninety samples were positive. Serum samples were tested for BDV-specific antibodies using a commercial ELISA kit and showed an average positive rate of 61.22% (30/49). Some individuals were identified as PI animals [91]. In 2017, a study detected BDV in Tibetan sheep samples in Haibei city of Qinghai Province, and the positive rates in 161 healthy sheep blood samples and 34 diarrhea sheep samples were 11.80% and 26.47%, respectively. In addition, 4.35% and 17.65% of healthy and diarrheal sheep samples showed coinfection of BDV and BVDV, respectively [92]. In another study, researchers examined fecal samples from Tibetan sheep with diarrhea collected from six counties in Haidong city of Qinghai Province during 2016–2017, and 23.96% of the samples (23/96) were positive for BDV [93]. In 2015, BDV-3 was identified from a sheep nasal swab sample in Shandong Province, and the sequence of the virus showed the highest phylogenetic relationship with JSLS12-01 [94]. Overall, epidemiological surveys of BDV have rarely been performed in China (Figure 2), and the prevalence and pathology of BDV infection under field conditions require further study.

Several studies have reported the adaptive plasticity of BDV to cross the species barrier and infect pigs and cattle [95,96,97,98,99,100,101]. Moreover, BDV can be transmitted among domesticated species and from domesticated to wild species, such as chamois, llama, alpaca, bison, and reindeer [102,103]. In one of our previous studies, the BDV strain JSLS12-01 of sheep origin successfully infected piglets and downregulated the antibody responses induced by the CSFV C strain [104]. Recently, BDV was isolated from *Melophagus ovinus* collected from the body surface of sheep in the Xinjiang Uygur Autonomous Region of China, emphasizing the potential role of this blood-sucking ectoparasite as a carrier of BDV [105]. Interspecies transmission of BDV in cattle and pigs might pose a significant challenge to the diagnosis and eradication of BVD and CSF. Therefore, when the surveillance of BDV is performed in a region, other related animal species should also be included simultaneously.

### 4.3. Diagnosis

Many methods have been used for the diagnosis of BDV infection, especially in PI animals. These methods include virus isolation, VNT, immunohistochemistry (IHC), ELISA, RT–PCR, and RT–qPCR [84,97,106,107]. ELISA and VNT are frequently used for serological investigations, while virus isolation, IHC, and nucleotide acid detection assays (RT–PCR, nRT-PCR, and qRT–PCR) are always used for agent examinations [86]. The methods and primers reported for BDV detection are summarized in Table 4. Furthermore, nucleotide acid sequencing plus phylogenetic analysis of RT–PCR products is frequently used to genotype clinical BDV strains. VNT is commonly used but is labor-intensive and time-consuming and thus is not suitable for the detection of large numbers of samples. An analysis of the cytopathic effect of BDV strains is recommended because of the simplicity of operation. No commercial BDV-specific ELISA kit is currently available in China. An ELISA kit that specifically detects antibodies targeting the p80/125 protein common to all BVDV and BDV strains (e.g., IDEXX) can be used. Furthermore, due to the cross-reactivity between antibodies against different *Pestivirus* members, a differential VNT is recommended in which sera are titrated against different *Pestivirus* members (BDV, BVDV-1, and BVDV-2). In general, virus isolation and VNT are technically demanding, labor-intensive and time-consuming methods. In clinical practice, nucleotide acid detection methods might be the first choice [108].

### 4.4. Prevention and Control

With the rapid development of the sheep and goat industry in China, a focus on the prevention and control of BDV infection is urgently needed. First, an understanding of the serological background is needed, and the lack of PI animals must be confirmed within the herds to eliminate the source of the infection. Treatment strategies may be another method to consider for BD prevention and control [108], but they are not recommended. The farm and/or farmers should focus on the quarantine and surveillance of BDV infection and the implementation of an eradication program. The two important diseases caused by Pestivirus (CSFV and BVDV) have been successfully controlled or eradicated in several countries, and effective strategies can be developed.

## 5. Other Emerging Viral Pathogens

### 5.1. Enzootic asal Tumor Virus (ENTV)

#### 5.1.1. Etiology

Enzootic nasal adenocarcinoma (ENA) is a contagious tumor of sheep and goats that is associated with airborne infection with ENTV [114]. ENTV belongs to the genus *Betaretrovirus* in the family *Retroviridae*. The ENTV genome is a single, positive-stranded RNA of 7.5 kb with a retroviral genome structure, 5′-U5-*gag-pro-pol-env*-U3-3′, consisting of four open reading frames (ORFs) and flanking untranslated regions and terminal repeats (LTRs) [115]. ENTV is genetically similar to jaagsiekte sheep retrovirus (JSRV), the causative virus of ovine pulmonary adenocarcinoma (OPA). Two types of ENTV have been identified: ENTV-1 in sheep and ENTV-2 in goats [116,117]. Genome sequence studies indicated that ENTV-1, ENTV-2, and JSRV are very similar but distinct from each other [118,119]. To date, 26 full-length sequences are available for ENTV-2; among them, 24 sequences have been obtained from Chinese strains. The genome length of these 26 isolates ranges from 7225 nt to 7503 nt (https://www.ncbi.nlm.nih.gov/, accessed on 10 May 2022).

ENTV-infected goats usually show copious serous nasal discharge, snuffling, and exophthalmos and progressively develop dyspnea and skull deformations. ENA is a chronic wasting disease, and the infected animals become emaciated and eventually die due to septicemic or toxemic complications. The duration of ENA from the appearance of clinical signs to death ranges from 3 weeks to 9 months or more [114,120]. The detailed pathology and pathogenesis of ENTV have been reviewed by De las Heras et al. [118].

#### 5.1.2. Epidemiology

ENA has been recorded in the majority of the countries where sheep and goats are farmed, except for Australia, New Zealand, and the UK [118]. The prevalence rate reaches 10% in some areas [121]. The first case of ENA was reported in Inner Mongolia, China, in 1995 [122]. Since then, ENA has not been examined and reported. During the last decade, ENA in goats has been reported in different provinces of China, including Hunan, Sichuan, Anhui, Shaanxi, Chongqing, Guizhou, Guangxi, Fujian, and Guangdong, which are the main breeding regions of goats (Figure 2). The viral genome sequences isolated from different provinces have been obtained and analyzed, which enriches the genetic information of ENTV-2 and will be helpful for further understanding the geographical distribution and evolutionary characteristics of ENTV-2. In addition, ENTV-1 infection in sheep has not been reported, suggesting a potential outbreak of ENTV-2 in China [7,123,124,125,126,127,128,129,130]. As OPA and JSRV have been reported in different sheep breeding regions in China, researchers should focus on understanding ENTV infection in both goats and sheep.

#### 5.1.3. Diagnosis

The diagnosis of ENA is routinely accomplished through epidemiological analysis, observation of specific clinical signs, and pathological findings at necropsy (nasal adenocarcinoma in the nasal cavity). The tumor arises from the ethmoidal mucosa and is gray or reddish-white in color, with a multilobular, granular, or soft surface [115,118,128]. The gross pathology and histopathology of ENA in sheep and goats are identical.

Due to the lack of evidence for circulating antibodies reported for ENA, the method of serological screening is not suitable for ENTV detection and ENA diagnosis [119,124,131]. Studies have concluded that ELISA and VNT showed low specificity and sensitivity in detecting experimental and field ENTV-1 infection [120,132]. In addition, Western blot also fails to detect any antibody against viral antigens from concentrated ENA fluid [118].

Interestingly, mAbs against the surface domain of the JSRV Env protein show good reactivity with tumor samples from JSRV-infected sheep and natural cases of ENA. These results imply that antibodies generated against Env proteins of JSRV and/or ENTV may serve as useful diagnostic tools for viral antigen detection using immunohistochemical (IHC) staining of contagious respiratory tumors from sheep and goats [133].

With the development of molecular biology technology and the availability of more information on ENTV genome sequences, highly sensitive, specific, and cost-effective conventional PCR or real-time PCR (qPCR) methods have been developed and widely used for the rapid detection of ENTV [115,134,135]. For ENTV-1, specific PCR and RT–PCR amplifying LTR and U5-*gag* regions have been developed [117], respectively (Table 5). Compared with ENTV-1, more detection methods, including PCR/RT–PCR and SYBR Green-, EvaGreen- and TaqMan-based qPCR/RT–qPCR, have been reported for ENTV-2. In these methods, *gag* and *env* were the two major target genes (Table 5). Two different protocols were employed: PCR and qPCR assays for the detection of viral RNA and RT–PCR and RT–qPCR assays for the detection of proviral DNA. During clinical application, PCR and qPCR have been used for neoplastic tissue samples, whereas RT–PCR and RT–qPCR might be applied for nasal discharge samples.

### 5.2. Caprine Herpesvirus 1 (CpHV-1)

CpHV-1 is a member of the alpha subfamily of the family *Herpesviridae*, which is genetically and antigenically related to bovine alphaherpesvirus 1 (BoHV-1) [139,140]. The virus was first isolated from goats in 1974 and further characterized in 1975 [141]. CpHV-1 infects goats through the respiratory and reproductive route, while displaying high tropism to the genital tract [142]. CpHV-1 infection usually causes systemic disease in young kids with high morbidity and mortality, while in adult goats, vulvovaginitis, balanoposthitis, respiratory disease, and occasionally abortions are induced [143,144,145]. CpHV-1 was reported in goat populations worldwide, including in the USA, Australia, New Zealand, Canada, Brazil, and Mediterranean countries (such as Greece, Italy, Spain, and France) [143,144,146,147].

The virus was first detected in goats suffering severe respiratory diseases in Jiangsu Province of China during 2013–2014, with a prevalence rate of 21.1% (40/190). A total of three different CpHV-1 strains were identified. Pathogenicity testing further confirmed that the JSHA1405 strain induced high fever and nasal discharges in infected goats [5]. A high seropositive rate (35/91) was found for the serum samples as examined by VNT [5]. In 2019, for the first time, the complete viral genome of strain JSHA1405 was identified. The viral genome is 134,617 bp in length with an average G+C content of 74.16% (GenBank accession number MG989243). A total of 69 open reading frames (ORFs) were predicted and annotated. Compared to BoHV-1, three ORFs (UL26.5, UL0.7, US1.67) were missing. Similar to other alphaherpesviruses, the UL region of the CpHV-1 genome encoded 57 ORFs and the US region encoded seven putative genes (US2 to US4 and US6 to US9) [5]. To date, no other report about CpHV-1 is available in China; the prevalence of the virus and its pathogenic role need to be investigated further in the future.

### 5.3. Enterovirus

The genus *Enterovirus* of the family *Picornaviridae* includes 9 species of enteroviruses (EV-A to EV-J) and 3 species of rhinoviruses (*Rhinovirus* A-C) [148]. EV-E, EV-F, and EV-G are associated with diseases of the livestock industry. EV-E and EV-F mainly infect cattle, and EV-G is responsible for swine enterovirus infections [6,149]. Enterovirus infections in animals in China have been investigated during the last decade, and enterovirus infections in goats were first reported in 2017 [150]. The first caprine enterovirus (CEV-JL14) was closely related to EV-G and was further designated EV-G20, although it seems to form a separate lineage in a phylogenetic analysis [151]. In 2019, another two novel enteroviruses were identified from a goat farm; one was classified as EV-F, while the other strain was clustered together with CEV-JL14 and an ovine enterovirus (TB4-OEV) [152]. Enterovirus infection in ruminants has rarely been reported in China, and more caprine/ovine enteroviruses need to be isolated and sequenced to better understand the prevalence of different enterovirus species in small ruminants and to categorize them.

Based on the sequence information from the isolated CEVs, a specific RT–PCR assay was developed and used for an epidemiological investigation [153]. In 2017-2019, 266 fecal samples collected from goats/sheep in Hebei Province showed an average positive rate of 27.82% [154]. CEV was detected in fecal samples from Tibetan sheep with diarrhea in Haidong city of Qinghai Province, suggesting a positive rate of 13.54% [93].

Furthermore, mAbs against viral VP1 protein were prepared and a sandwich ELISA was developed for more efficient screening of CEV antigen from fecal samples [155]. A high positive rate was found in Henan (40.27%, 120/298) and Shandong (52.75%, 96/182) provinces. The positive rate was significantly higher than that in healthy herds (12.11%, 94/776) [156,157].

## 6. Conclusions and Future Perspectives

With the rapid development of the intensive sheep/goat raising industry in recent years, frequent allocation and transportation, and poor disease control strategies in some farms, several emerging pathogens have been introduced and are prevalent in sheep and/or goat herds in China, resulting in varying degrees of economic losses. Some viruses, such as PPRV, cause direct damage to animals and have received more attention from veterinarians and researchers. Others, such as CPIV3, BDV, enterovirus, and CpHV-1, usually induce subclinical or mild diseases and have not been reported frequently, and thus they are still underestimated or neglected.

For PPR, compulsory immunization has been conducted for many years, and it has been well controlled, although it still occurs sporadically. Next, farmers and researchers should focus on the evaluation of vaccination and novel DIVA vaccine development. Regional eradication programs should be established to achieve PPR eradication in China.

Regarding the other emerging viruses, first, they deserve more attention. Second, epidemiological investigations should be continuously performed, especially for the major breeding regions and suspected diseased herds, to further understand the infection status of these viruses and the relationship of viral infection with clinical diseases. Investigations of the prevalence of BDV and BVDV in small ruminants are also very important for the diagnosis and eradication of BVDV and CSFV in cattle and pigs, respectively. The distribution of EV and CPIV3 and the mixed infection status of these viruses with other pathogens requires further study. Third, different prevention strategies should be implemented for different pathogens. Early prevention, accurate diagnosis, and supportive treatment are the key goals. In particular, no vaccine or effective treatment methods are available for BDV, CpHV-1, and ENTV, and biosecurity strategies, including quarantine, disinfection, and “all-in all-out” procedures are powerful tools.

Furthermore, the epidemic dynamics of other foreign diseases and related pathogens, such as Rift Valley fever virus (RVFV), other serotypes of bluetongue virus (BTV) and foot and mouth disease virus (FMDV), and ENTV-2, should receive more attention. More efforts are needed to develop dependable diagnostic methods and formulate an epidemic prevention policy for these viruses to prevent their introduction.

## Figures and Tables

**Table 1:** Summary of PPRV and IFN pathway interactions.

Interferon Type	Key Points or Pathways	Related Viral Proteins	References
type I IFN pathways	MAVS	/	[20]
type I interferon	IRF3, TBK1–IRF3 complex	N	[24]
IFN-λ3, IFN-β, and IFN-λ2	RIG-I-like receptor, IRF3	P	[25]
IFN-β	JAK–STAT, MAVS, and RIG-I signaling pathway	C	[26]
IFN-β, IFN-γ	STAT1	N and P	[23]
IFN	ATG13	/	[21]
type I IFN pathway	miR-3	/	[27]

**Table 2:** Sero-epidemiological study against CPIV3 in different regions of China.

Region	Province	Positive No./Total No. Positive Rate (%)		Region	Province	Positive No./Total No. Positive Rate (%)	
		Goat	Sheep			Goat	Sheep
East China	Jiangsu	1137/2842 40.01	356/1078 33.02	Northwest China	Xinjiang	/	408/568 71.83
	Anhui	273/658 41.49	24/219 10.96		Qinghai	/	73/118 61.86
	Shandong	162/855 ^b^ 18.95	342/728 46.98		Gansu	/	592/848 69.81
	Zhejiang	31/98 31.63	/		Shannxi	66/254 25.98	/
North China	Inner Mongolia	/	89/105 84.76	Southwest China	Tibet	/	12/104 12.54
	Hebei	/	150/332 45.18		Guizhou	46/158 29.11	/
	Shanxi	39/93 41.94	/	Northeast China	Jilin	173/480 36.04	/
South China	Guangdong	0/200 0	/		Liaoning	38/167 22.75	/
	Guangxi	171/634 ^a^ 26.97	/				

^a^ Including the result from Bi, J.S., et al. [69]. ^b^ The result from Chen, J.L., et al. [70]. / = not tested.

**Table 3:** Nucleotide acid detection methods used for CPIV3 diagnosis.

Methods	Primer	Sequence (5′-3′)	Size	Reference
RT-PCR	F	AGTGATCTAGATGATGATCCA	329bp	[79]
	R	GTTATTGATCCAATTGCTGT		
RT-PCR	F	GCAATCCACCAAAGCATGGGGT	346bp	[80]
	R	GGGGCAAGTGCTACTTTTTGAGCA		
RT-qPCR	qF	GCTTGGCTTCTTTGAAATGG	150bp	[67]
	qR	GCCTGCAGAAGTTCCTTGTC		
	Probe	FAM-CAATCGGACTAGCCAAGTATGGTGGGA-TAMRA		

**Table 4:** Reported primers that can be used for BDV detection.

Methods	Primers	Sequences (5′-3′)	Target	Size (bp)	Reference
RT-PCR	324	ATGCCCWTAGTAGGACTAGCA	5′-UTR	288	[109]
	326	TCAACTCCATGTGCCATGTAC			
RT-PCR	PBD1	TCGTGGTGAGATCCCTGAG	5′-UTR	225	[110]
	PBD2	GCAGAGATTTTTTATACTAGCCTATRC			
RT-PCR	320F	GCCTGATAGGGTGYWGCAGAG	N^pro^-C	740	[111]
	1040R	TTYCCTTTCTTCTTYACCTGGTA			
RT-PCR	BD1	TCTCTGCTGTACATGGCACATG	N^pro^-C	738	[112]
	BD2	TTGTTRTGGTACARRCCGTC			
RT-qPCR (Probe)	BDV87F	CCGTGTTAACCATACACGTAGTAGGA	5′-UTR	155	[113]
	BDV237	GCCCTCGTCCACGTAGCA			
	BDV136T (probe)	VIC-CTCAGGGATCTCACCACGA-NFQ-MGB			

**Table 5:** PCR methods developed for ENTV detection.

Method	Targets	Primer	Sequence (5′-3′)	References
RT-PCR	ENTV-1U5-*gag*	F	GATGCTCCGTTCTCTCCTTATA	[120]
		R	GGGACGCGACGAATGTAGG	
PCR	ENTV-1LTR	F	AAGCAAGTTAAGTAACTTGAGATC	[117]
		R	GCTTAGCCGTCCTAAAAGAG	
PCR	ENTV-2*env*	F	AGCTGCTCATACTGTGGATC	
		R	GATCTTATCTGCTTATTTTCAG	
RT-PCR	ENTV-2*gag*	F	AAATGCGACCTTCCGATAATGATGA	[135]
		R	CTTCTGTAGCGGGGACATATTCTCA	
PCR	ENTV-2*gag*	F	GTCCCTAAAAATGCGACCTT	[136]
		R	GCGACTCCTGAGTTCTGTAAAACCAC	
qPCR/RT-qPCR (Probe)	ENTV-2*env*-U3	F	CCTAACCTTCAT TCRTTATGGCARAGT	[115]
		R	CACCGGATCCTTAYGTAATCRGATTTCCTG	
		Probe	FAM-TGTTTAGTTCCTTGCCTCCTTCGTGG-IBFQ	
qPCR (SYBR Green)	ENTV-2LTR	F	GAGATTTCTTACACATGAGAGC	[137]
		R	TCCCAGGACTTAACCATTC	
qPCR (Eva Green)	ENTV-2*env*	F	GAGGCAAATTGAGGCGTTGAT	[134]
		R	CCCGTTCTGCATTCGCTGTAG	
qPCR (Probe)	ENTV-2*env*	F	ATGGCAATAGTTTATATCTGCAAT	[138]
		R	GATGGCCTTGTATCAACATAAATGG	
		Probe	FAM-ATATAAGAATCCCGTAACACCTACATCTC-BHQ1	

## Data Availability

Not applicable.

## References

[1] Liu F., Li J., Li L., Liu Y., Wu X., Wang Z. (2018). Peste des petits ruminants in China since its first outbreak in 2007: A 10-year review. Transbound. Emerg. Dis..

[2] Li W., Mao L., Cheng S., Wang Q., Huang J., Deng J., Wang Z., Zhang W., Yang L., Hao F. (2014). A novel parainfluenza virus type 3 (PIV3) identified from goat herds with respiratory diseases in eastern China. Vet. Microbiol..

[3] Mao L., Liu X., Li W., Yang L., Zhang W., Jiang J. (2015). Characterization of one sheep border disease virus in China. Virol. J..

[4] Li W., Mao L., Zhao Y., Sun Y., He K., Jiang J. (2013). Detection of border disease virus (BDV) in goat herds suffering diarrhea in eastern China. Virol. J..

[5] Hao F., Mao L., Li W., Li J., Yang L., Zhang W., Jiang J., Sun M., Xie X., Liu M. (2020). Epidemiological investigation and genomic characterization of Caprine herpesvirus 1 from goats in China. Infect. Genet. Evol. J. Mol. Epidemiol. Evol. Genet. Infect. Dis..

[6] Zhu L., Xing Z., Gai X., Li S., San Z., Wang X. (2014). Identification of a novel enterovirus E isolates HY12 from cattle with severe respiratory and enteric diseases. PLoS ONE.

[7] Yi G., Kaiyu W., Qigui Y., Zhongqiong Y., Yingdong Y., Defang C., Jinlu H. (2010). Descriptive study of enzootic nasal adenocarcinoma in goats in southwestern China. Transbound. Emerg. Dis..

[8] Adams M.J., Lefkowitz E.J., King A.M.Q., Harrach B., Harrison R.L., Knowles N.J., Kropinski A.M., Krupovic M., Kuhn J.H., Mushegian A.R. (2017). Changes to taxonomy and the International Code of Virus Classification and Nomenclature ratified by the International Committee on Taxonomy of Viruses (2017). Arch. Virol..

[9] FAO, OIE (2015). Global Control and Eradication of Peste Des Petits Ruminants.

[10] Wang Z.L., Bao J.Y., Wu X.D., Liu Y.T., Li L., Liu P.L., Zhao Y.G., Liu C.J., Xiao X. (2007). Diagnosis of the first outbreak of peste des petits ruminants in Tibet. China Anim. Health Insp..

[11] Wang Z., Bao J., Wu X., Liu Y., Li L., Liu C., Suo L., Xie Z., Zhao W., Zhang W. (2009). Peste des petits ruminants virus in Tibet, China. Emerg. Infect. Dis..

[12] Xia J., Zheng X.G., Adili G.Z., Wei Y.R., Ma W.G., Xue X.M., Mi X.Y., Yi Z., Chen S.J., Du W. (2016). Sequence analysis of peste des petits ruminants virus from ibexes in Xinjiang, China. Genet. Mol. Res. GMR.

[13] Bao J., Wang Q., Li L., Liu C., Zhang Z., Li J., Wang S., Wu X., Wang Z. (2017). Evolutionary dynamics of recent peste des petits ruminants virus epidemic in China during 2013–2014. Virology.

[14] Bao J., Wang Q., Zhang Y., Liu C., Li L., Wang Z. (2014). Complete Genome Sequence of a Novel Variant Strain of Peste des Petits Ruminants Virus, China/XJYL/2013. Genome Announc..

[15] Li J., Li L., Wu X., Liu F., Zou Y., Wang Q., Liu C., Bao J., Wang W., Ma W. (2017). Diagnosis of Peste des Petits Ruminants in Wild and Domestic Animals in Xinjiang, China, 2013-2016. Transbound. Emerg. Dis..

[16] Zhu Z., Zhang X., Adili G., Huang J., Du X., Zhang X., Li P., Zheng X., Liu X., Zheng H. (2016). Genetic Characterization of a Novel Mutant of Peste Des Petits Ruminants Virus Isolated from Capra ibex in China during 2015. BioMed Res. Int..

[17] Li L., Cao X., Wu J., Dou Y., Meng X., Liu D., Liu Y., Shang Y., Liu X. (2019). Epidemic and evolutionary characteristics of peste des petits ruminants virus infecting Procapra przewalskii in Western China. Infect. Genet. Evol. J. Mol. Epidemiol. Evol. Genet. Infect. Dis..

[18] Ying L., Yang Y.Q., Li L., Zhao Q.B., Wang X.Z., La H., Liu F.X., Tan S.K., Song L.Z., Wu X.D. (2019). Diagnosis and control of the first peste des petits ruminants infecting wild Pseudois nayaur in the Qinghai plateau area. Anim. Husb. Vet. Med..

[19] Ma J., Gao X., Liu B., Chen H., Xiao J., Wang H. (2019). Peste des petits ruminants in China: Spatial risk analysis. Transbound. Emerg. Dis..

[20] Miao Q., Qi R., Meng C., Zhu J., Tang A., Dong D., Guo H., van Oers M.M., Pijlman G.P., Liu G. (2021). Caprine MAVS Is a RIG-I Interacting Type I Interferon Inducer Downregulated by Peste des Petits Ruminants Virus Infection. Viruses.

[21] Ma P., Li L., Jin L., Zhang D., Cao X., Guo F., Zhao Y., Bai J., Ma Z., Shang Y. (2020). Antiviral responses of ATG13 to the infection of peste des petits ruminants virus through activation of interferon response. Gene.

[22] Chen S., Yang F., Cao W., Liu H., Wen B., Sun Y., Zheng H., Wang J., Zhu Z. (2021). Quantitative Proteomics Reveals a Novel Role of the E3 Ubiquitin-Protein Ligase FANCL in the Activation of the Innate Immune Response through Regulation of TBK1 Phosphorylation during Peste des Petits Ruminants Virus Infection. J. Proteome Res..

[23] Li P., Zhu Z., Zhang X., Dang W., Li L., Du X., Zhang M., Wu C., Xue Q., Liu X. (2019). The Nucleoprotein and Phosphoprotein of Peste des Petits Ruminants Virus Inhibit Interferons Signaling by Blocking the JAK-STAT Pathway. Viruses.

[24] Zhu Z., Li P., Yang F., Cao W., Zhang X., Dang W., Ma X., Tian H., Zhang K., Zhang M. (2019). Peste des Petits Ruminants Virus Nucleocapsid Protein Inhibits Beta Interferon Production by Interacting with IRF3 To Block Its Activation. J. Virol..

[25] Li P., Zhu Z., Cao W., Yang F., Ma X., Tian H., Zhang K., Liu X., Zheng H. (2021). Dysregulation of the RIG-I-like Receptor Pathway Signaling by Peste des Petits Ruminants Virus Phosphoprotein. J. Immunol..

[26] Li L., Shi X., Ma X., Cao X., Ali A., Bai J. (2021). Peste des petits ruminants virus non-structural C protein inhibits the induction of interferon-beta by potentially interacting with MAVS and RIG-I. Virus Genes.

[27] Li H., Xue Q., Wan Y., Chen Y., Zeng W., Wei S., Zhang Y., Wang J., Qi X. (2021). PPRV-induced novel miR-3 contributes to inhibit type I IFN production by targeting IRAK1. J. Virol..

[28] Zhang Y., Wu S., Lv J., Feng C., Deng J., Wang C., Yuan X., Zhang T., Lin X. (2013). Peste des petits ruminants virus exploits cellular autophagy machinery for replication. Virology.

[29] Yang B., Xue Q., Guo J., Wang X., Zhang Y., Guo K., Li W., Chen S., Xue T., Qi X. (2020). Autophagy induction by the pathogen receptor NECTIN4 and sustained autophagy contribute to peste des petits ruminants virus infectivity. Autophagy.

[30] Yang B., Xue Q., Qi X., Wang X., Jia P., Chen S., Wang T., Xue T., Wang J. (2018). Autophagy enhances the replication of Peste des petits ruminants virus and inhibits caspase-dependent apoptosis in vitro. Virulence.

[31] Wan Y., Chen Y., Wang T., Zhao B., Zeng W., Zhang L., Zhang Y., Cao S., Wang J., Xue Q. (2022). PPRV-Induced Autophagy Facilitates Infectious Virus Transmission by the Exosomal Pathway. J. Virol..

[32] Xue Q., Liu H., Sun M., Zhao W., Chen Y., Chen J., Wei C., Cai X., Xue Q. (2020). Peste des petits ruminants virus hemagglutinin (H) induces lysosomal degradation of host cyclophilin A to facilitate viral replication. Virus Res..

[33] Li L., Yang W., Ma X., Wu J., Qin X., Cao X., Zhou J., Jin L., He J., Zheng H. (2022). Peste Des Petits Ruminants Virus N Protein Is a Critical Proinflammation Factor That Promotes MyD88 and NLRP3 Complex Assembly. J. Virol..

[34] Qi X., Li Z., Li H., Wang T., Zhang Y., Wang J. (2019). MicroRNA-1 Negatively Regulates Peripheral NK Cell Function via Tumor Necrosis Factor-Like Weak Inducer of Apoptosis (TWEAK) Signaling Pathways During PPRV Infection. Front. Immunol..

[35] Kinimi E., Odongo S., Muyldermans S., Kock R., Misinzo G. (2020). Paradigm shift in the diagnosis of peste des petits ruminants: Scoping review. Acta Vet. Scand..

[36] Li L., Wu X., Liu F., Wang Z., Liu C., Wang Q., Bao J. (2016). Rapid detection of lineage IV peste des petits ruminants virus by real-time RT-PCR. J. Virol. Methods.

[37] Li L., Bao J., Wu X., Wang Z., Wang J., Gong M., Liu C., Li J. (2010). Rapid detection of peste des petits ruminants virus by a reverse transcription loop-mediated isothermal amplification assay. J. Virol. Methods.

[38] Yang Y., Qin X., Song Y., Zhang W., Hu G., Dou Y., Li Y., Zhang Z. (2017). Development of real-time and lateral flow strip reverse transcription recombinase polymerase Amplification assays for rapid detection of peste des petits ruminants virus. Virol. J..

[39] Li Y., Li L., Fan X., Zou Y., Zhang Y., Wang Q., Sun C., Pan S., Wu X., Wang Z. (2018). Development of real-time reverse transcription recombinase polymerase amplification (RPA) for rapid detection of peste des petits ruminants virus in clinical samples and its comparison with real-time PCR test. Sci. Rep..

[40] Zhang Y., Wang J., Zhang Z., Mei L., Wang J., Wu S., Lin X. (2018). Development of recombinase polymerase amplification assays for the rapid detection of peste des petits ruminants virus. J. Virol. Methods.

[41] Yang M.F., Ma Y.Y., Yu Z.Y., Liu J.S., Kang H.T., Jiang Q., Qu L.D. (2021). Development and application of a chip digital PCR assay for PPRV. China Anim. Health Insp..

[42] Prajapati M., Dou Y., Zhu X., Zhao S., Alfred N., Li Y., Zhang Z. (2020). Development of an Enzyme-Linked Immunosorbent Assay Based on CD150/SLAM for the Detection of Peste des Petits Ruminant Virus. Front. Vet. Sci..

[43] Ruan Z.Y., Hu B., Song Y.H., Wei H.J., Fan Z.Y., Wu X.D., Wang F. (2018). Establishment and preliminary application of a simplex suspension array for detection of the peste des petits ruminants virus. Anim. Husb. Vet. Med..

[44] Sun Y., Song X., Xiao Y., Li X., Lv Y., Wang R., Jiang F., Sun H., Yang L., Wang C. (2019). A comparative study on double-antigen S-ELISA for the detections of antibodies based on different recombinant N protein of peste des petits ruminants virus. Chin. Vet. Sci..

[45] Qian B., Li Y.M., Zhu X.L., Y Z.X., Niyokwishimira A., Dou Y.X., Zhang Z.D. (2021). Establishment of an iELISA method for detection of antibody against Peste des petits ruminants virus (PPRV) based on H protein epitope peptide. Acta Vet. Et Zootech. Sin..

[46] Zhang G.R., Yu R.S., Zeng J.Y., Zhu Y.M., Dong S.J., Dunzhu L., Zhu S., Duoji C., Lei Z.H., Li Z. (2013). Development of an epitope-based competitive ELISA for the detection of antibodies against Tibetan peste des petits ruminants virus. Intervirology.

[47] Cheng S., Sun J., Yang J., Lv J., Wu F., Lin Y., Liao L., Ye Y., Cao C., Fang L. (2017). A new immunoassay of serum antibodies against Peste des petits ruminants virus using quantum dots and a lateral-flow test strip. Anal. Bioanal. Chem..

[48] Sun Y., Song X.H., Xiao Y., Wang R.N., Li X.M., Ren X.J., Li X.G., Wei W., Yang L., Wang C.B. (2020). Establishment of chemiluminescent immunoassay for detection of antibody against peste des petits ruminant virus. Anim. Husb. Vet. Med..

[49] Hodgson S., Moffat K., Hill H., Flannery J.T., Graham S.P., Baron M.D., Darpel K.E. (2018). Comparison of the Immunogenicities and Cross-Lineage Efficacies of Live Attenuated Peste des Petits Ruminants Virus Vaccines PPRV/Nigeria/75/1 and PPRV/Sungri/96. J. Virol..

[50] Hao F., Li W.L., Mao L., Li J.Z., Yang L.L., Zhang W.W., Sun M., Liu M.J., Jiang J.Y. (2018). Influence of PPR maternal antibody and vaccination of goat pox vaccine and FMD vaccine on efficiency of PPR vaccine. Chin. J. Vet. Sci..

[51] Chen W., Hu S., Qu L., Hu Q., Zhang Q., Zhi H., Huang K., Bu Z. (2010). A goat poxvirus-vectored peste-des-petits-ruminants vaccine induces long-lasting neutralization antibody to high levels in goats and sheep. Vaccine.

[52] Hu Q., Chen W., Huang K., Baron M.D., Bu Z. (2012). Rescue of recombinant peste des petits ruminants virus: Creation of a GFP-expressing virus and application in rapid virus neutralization test. Vet. Res..

[53] Yin C., Chen W., Hu Q., Wen Z., Wang X., Ge J., Yin Q., Zhi H., Xia C., Bu Z. (2014). Induction of protective immune response against both PPRV and FMDV by a novel recombinant PPRV expressing FMDV VP1. Vet. Res..

[54] Wang Y., Liu G., Shi L., Li W., Li C., Chen Z., Jin H., Xu B., Li G. (2013). Immune responses in mice vaccinated with a suicidal DNA vaccine expressing the hemagglutinin glycoprotein from the peste des petits ruminants virus. J. Virol. Methods.

[55] Li W., Jin H., Sui X., Zhao Z., Yang C., Wang W., Li J., Li G. (2014). Self-assembly and release of peste des petits ruminants virus-like particles in an insect cell-baculovirus system and their immunogenicity in mice and goats. PLoS ONE.

[56] Yan F., Banadyga L., Zhao Y., Zhao Z., Schiffman Z., Huang P., Li E., Wang C., Gao Y., Feng N. (2019). Peste des Petits Ruminants Virus-Like Particles Induce a Potent Humoral and Cellular Immune Response in Goats. Viruses.

[57] Yan F., Li E., Li L., Schiffman Z., Huang P., Zhang S., Li G., Jin H., Wang H., Zhang X. (2020). Virus-Like Particles Derived From a Virulent Strain of Pest des Petits Ruminants Virus Elicit a More Vigorous Immune Response in Mice and Small Ruminants Than Those From a Vaccine Strain. Front. Microbiol..

[58] Liu F., Wu X., Zou Y., Li L., Wang Z. (2015). Peste des petits ruminants virus-like particles induce both complete virus-specific antibodies and virus neutralizing antibodies in mice. J. Virol. Methods.

[59] Njeumi F., Bailey D., Soula J.J., Diop B., Tekola B.G. (2020). Eradicating the Scourge of Peste Des Petits Ruminants from the World. Viruses.

[60] Wu H., ManChu R., Wang F., Li C., Jin N., Chen Q. (2022). Epidimiological situation of research progress on molecular detection of peste des petits ruminants virus. Prog. Vet. Med..

[61] Yang L., Li W., Mao L., Hao F., Wang Z., Zhang W., Deng J., Jiang J. (2016). Analysis on the complete genome of a novel caprine parainfluenza virus 3. Infect. Genet. Evol. J. Mol. Epidemiol. Evol. Genet. Infect. Dis..

[62] Makoschey B., Berge A.C. (2021). Review on bovine respiratory syncytial virus and bovine parainfluenza-usual suspects in bovine respiratory disease-a narrative review. BMC Vet. Res..

[63] Ma Y., Wang Y., Zan X., Wu Y., Wang J., Li G., Chai C., Fu C., Wang S., Yin H. (2021). Phylogenetic and pathogenicity analysis of a novel lineage of caprine parainfluenza virus type 3. Microb. Pathog..

[64] Zhu Y.M., Chen R.H., Lin J., Yang M.J., Xue F. (2020). Isolation and identification of ovine parainfluenza virus type 3 strain PE2019. Chin. J. Prev. Vet. Med..

[65] Mao L., Yang L., Li W., Liang P., Zhang S., Li J., Sun M., Zhang W., Wang L., Zhong C. (2019). Epidemiological investigation and phylogenetic analysis of caprine parainfluenza virus type 3 in sheep of China. Transbound. Emerg. Dis..

[66] Mao L., Li W., Zhou T., Yang L., Hao F., Li J., Zhang W., Luo X., Jiang J. (2017). Development of a blocking ELISA for Caprine parainfluenza virus type 3. J. Virol. Methods.

[67] Li J., Li W., Mao L., Hao F., Yang L., Zhang W., Jiang J. (2016). Rapid detection of novel caprine parainfluenza virus type 3 (CPIV3) using a TaqMan-based RT-qPCR. J. Virol. Methods.

[68] Li W., Hao F., Mao L., Wang Z., Zhou T., Deng J., Li J., Zhang W., Yang L., Lv Y. (2016). Pathogenicity and horizontal transmission studies of caprine parainfluenza virus type 3 JS2013 strain in goats. Virus Res..

[69] Bi J.S., Wang W.X., Wei X.K., Su J.X., Zheng M. (2018). Serological investigation of antibody against Caprine Parainfluenza virus type 3 in Guangxi. Heilongjiang Anim. Sci. Vet. Med..

[70] Chen J.L., Huang X.J., Hao S.N., Zhang D.P., Wang J.L., Shen Z.Q. (2020). Truncated expression of Caprine Parainfluenza virus type 3 NP protein and establishment of indirect ELISA. Chin. J. Vet. Sci..

[71] Li W., Yang L., Mao L., Liu M., Li J., Zhang W., Sun M. (2020). Cholesterol-rich lipid rafts both in cellular and viral membrane are critical for caprine parainfluenza virus type3 entry and infection in host cells. Vet. Microbiol..

[72] Li J., Mao L., Xiao F., Liao Z., Yin J., Li W., Sun M., Liu M., Ji X., Liu C. (2020). Interferon-stimulated genes inhibit caprine parainfluenza virus type 3 replication in Madin-Darby bovine kidney cells. Vet. Microbiol..

[73] Mao L., Liang P., Li W., Zhang S., Liu M., Yang L., Li J., Li H., Hao F., Sun M. (2020). Exosomes promote caprine parainfluenza virus type 3 infection by inhibiting autophagy. J. Gen. Virol..

[74] Li J., Yang L., Mao L., Li W., Sun M., Liu C., Xue T., Zhang W., Liu M., Li B. (2021). Caprine parainfluenza virus type 3 N protein promotes viral replication via inducing apoptosis. Vet. Microbiol..

[75] Li J., Zhong C., Liao Z., Mao L., Li W., Sun M., Liu M., Ji X., Liu C., Xue T. (2020). Bta-miR-98 Suppresses Replication of Caprine Parainfluenza Virus Type 3 Through Inhibiting Apoptosis by Targeting Caspase-3. Front. Immunol..

[76] Sun M., Li W., Zhang W., Yang L., Hao F., Li J., Mao L., Jiang J., Liu M. (2021). Screening interferon antagonists from accessory proteins encoded by P gene for immune escape of Caprine parainfluenza virus 3. Vet. Microbiol..

[77] Li J., Mao L., Zhong C., Li W., Hao F., Sun M., Zhu X., Ji X., Xiao F., Yang L. (2018). Cellular microRNA bta-miR-222 suppresses caprine parainfluenza virus type 3 replication via downregulation of interferon regulatory factor 2. Vet. Microbiol..

[78] Ellis J.A. (2010). Bovine parainfluenza-3 virus. Vet. Clin. N. Am. Food Anim. Pract..

[79] Maidana S.S., Lomonaco P.M., Combessies G., Craig M.I., Diodati J., Rodriguez D., Parreno V., Zabal O., Konrad J.L., Crudelli G. (2012). Isolation and characterization of bovine parainfluenza virus type 3 from water buffaloes (Bubalus bubalis) in Argentina. BMC Vet. Res..

[80] Yun J.L., He M.F., Wang S.H., Mao L., Yang L.L., Zhang W.W., Sun M., Liu M.J., Li W.L. (2022). Diagnosis and pathogenic identification of the respiratory disease in a fattening goat farm. Anim. Husb. Vet. Med..

[81] Wang M., Li W.L., Hao F., Mao L., Yang L.L., Zhang W.W., Jiang J.Y. (2017). Prokaryotic expression of N protein of Caprine Parainfluenza virus type 3 and establishment of indirect ELISA antibody detection method. Acta Vet. Et Zootech. Sin..

[82] Nettleton P.F., Gilray J.A., Russo P., Dlissi E. (1998). Border disease of sheep and goats. Vet. Res..

[83] Nettleton P.F., Entrican G. (1995). Ruminant pestiviruses. Br. Vet. J..

[84] Oguzoglu T.C., Tan M.T., Toplu N., Demir A.B., Bilge-Dagalp S., Karaoglu T., Ozkul A., Alkan F., Burgu I., Haas L. (2009). Border disease virus (BDV) infections of small ruminants in Turkey: A new BDV subgroup?. Vet. Microbiol..

[85] King A.M.Q., Lefkowitz E.J., Mushegian A.R., Adams M.J., Dutilh B.E., Gorbalenya A.E., Harrach B., Harrison R.L., Junglen S., Knowles N.J. (2018). Changes to taxonomy and the International Code of Virus Classification and Nomenclature ratified by the International Committee on Taxonomy of Viruses (2018). Arch. Virol..

[86] Righi C., Petrini S., Pierini I., Giammarioli M., De Mia G.M. (2021). Global Distribution and Genetic Heterogeneity of Border Disease Virus. Viruses.

[87] Peletto S., Caruso C., Cerutti F., Modesto P., Zoppi S., Dondo A., Acutis P.L., Masoero L. (2016). A new genotype of border disease virus with implications for molecular diagnostics. Arch. Virol..

[88] Giammarioli M., La Rocca S.A., Steinbach F., Casciari C., De Mia G.M. (2011). Genetic and antigenic typing of border disease virus (BDV) isolates from Italy reveals the existence of a novel BDV group. Vet. Microbiol..

[89] Sozzi E., Lavazza A., Gaffuri A., Bencetti F.C., Prosperi A., Lelli D., Chiapponi C., Moreno A. (2019). Isolation and Full-Length Sequence Analysis of a Pestivirus from Aborted Lamb Fetuses in Italy. Viruses.

[90] Casciari C., Sozzi E., Bazzucchi M., Moreno Martin A.M., Gaffuri A., Giammarioli M., Lavazza A., De Mia G.M. (2020). Serological relationship between a novel ovine pestivirus and classical swine fever virus. Transbound. Emerg. Dis..

[91] Li W.L., Mao L., Zhang W.W., Zhang Z.B., Zhao Y.Q., He K.W., Jiang J.Y. (2013). Isolation and identification of border disease virus (BDV) from a goat herd suffering unremitting diarrhea. Jiangsu J. Agric. Sci..

[92] Chen Y.Z., Bao G.C., Zhang S.X., Han M. (2017). Epidemiological investigation of bovine viral diarrhea virus and sheep boundary virus in Tibetan sheep in Haibei area, Qinghai province. Chin. J. Vet. Drug.

[93] Wang Y.L., Xu S.R., Zhang X.Z., Xu C.F. (2018). Etiological investigation and analysis of bovine viral diarrhea virus, border disease virus and enterovirus in Tibetan sheep in Haidong City, Qinghai Province. Anim. Husb. Vet. Med..

[94] Peng C., Wang S.C., Zhuang Q.Y., Hou G.Y., Huang J., Shan H., Chen J.M. (2015). Detection of border disease virus (BDV) in apparently healthy sheep in Shandong. China Anim. Health Insp..

[95] Vilcek S., Belak S. (1996). Genetic identification of pestivirus strain Frijters as a border disease virus from pigs. J. Virol. Methods.

[96] Nagai M., Aoki H., Sakoda Y., Kozasa T., Tominaga-Teshima K., Mine J., Abe Y., Tamura T., Kobayashi T., Nishine K. (2014). Molecular, biological, and antigenic characterization of a Border disease virus isolated from a pig during classical swine fever surveillance in Japan. J. Vet. Diagn. Investig. Off. Publ. Am. Assoc. Vet. Lab. Diagn. Inc..

[97] Kawanishi N., Tsuduku S., Shimizu H., Ohtani Y., Kameyama K., Yamakawa M., Tsutsui T., Matsuura K., Ohashi S., Isobe T. (2014). First isolation of border disease virus in Japan is from a pig farm with no ruminants. Vet. Microbiol..

[98] Rosell R., Cabezon O., Pujols J., Domingo M., Munoz I., Nunez J.I., Ganges L. (2014). Identification of a porcine pestivirus as a border disease virus from naturally infected pigs in Spain. Vet. Rec..

[99] Hornberg A., Fernandez S.R., Vogl C., Vilcek S., Matt M., Fink M., Kofer J., Schopf K. (2009). Genetic diversity of pestivirus isolates in cattle from Western Austria. Vet. Microbiol..

[100] Gomez-Romero N., Basurto-Alcantara F.J., Verdugo-Rodriguez A., Lagunes-Quintanilla R., Bauermann F.V., Ridpath J.F. (2018). Detection of border disease virus in Mexican cattle. Transbound. Emerg. Dis..

[101] Braun U., Hilbe M., Peterhans E., Schweizer M. (2019). Border disease in cattle. Vet. J..

[102] Becher P., Orlich M., Kosmidou A., Konig M., Baroth M., Thiel H.J. (1999). Genetic diversity of pestiviruses: Identification of novel groups and implications for classification. Virology.

[103] Martin C., Duquesne V., Adam G., Belleau E., Gauthier D., Champion J.L., Saegerman C., Thiery R., Dubois E. (2015). Pestiviruses infections at the wild and domestic ruminants interface in the French Southern Alps. Vet. Microbiol..

[104] Mao L., Li W., Liu X., Hao F., Yang L., Deng J., Zhang W., Wei J., Jiang J. (2015). Chinese border disease virus strain JSLS12-01 infects piglets and down-regulates the antibody responses of classical swine fever virus C strain vaccination. Vaccine.

[105] Liu Y.H., He B., Li K.R., Li F., Zhang L.Y., Li X.Q., Zhao L. (2019). First report of border disease virus in Melophagus ovinus (sheep ked) collected in Xinjiang, China. PLoS ONE.

[106] Marco I., Rosell R., Cabezon O., Beneria M., Mentaberre G., Casas E., Hurtado A., Lopez-Olvera J.R., Lavin S. (2009). Serologic and virologic investigations into pestivirus infection in wild and domestic ruminants in the Pyrenees (NE Spain). Res. Vet. Sci..

[107] Thabti F., Letellier C., Hammami S., Pepin M., Ribiere M., Mesplede A., Kerkhofs P., Russo P. (2005). Detection of a novel border disease virus subgroup in Tunisian sheep. Arch. Virol..

[108] Newcomer B.W., Givens M.D. (2013). Approved and experimental countermeasures against pestiviral diseases: Bovine viral diarrhea, classical swine fever and border disease. Antivir. Res..

[109] Vilcek S., Herring A.J., Herring J.A., Nettleton P.F., Lowings J.P., Paton D.J. (1994). Pestiviruses isolated from pigs, cattle and sheep can be allocated into at least three genogroups using polymerase chain reaction and restriction endonuclease analysis. Arch. Virol..

[110] Vilbek S., Paton D.J. (2000). A RT-PCR assay for the rapid recognition of border disease virus. Vet. Res..

[111] Strong R., La Rocca S.A., Ibata G., Sandvik T. (2010). Antigenic and genetic characterisation of border disease viruses isolated from UK cattle. Vet. Microbiol..

[112] Vilcek S., Nettleton P.F., Paton D.J., Belak S. (1997). Molecular characterization of ovine pestiviruses. J. Gen. Virol..

[113] Willoughby K., Valdazo-Gonzalez B., Maley M., Gilray J., Nettleton P.F. (2006). Development of a real time RT-PCR to detect and type ovine pestiviruses. J. Virol. Methods.

[114] De las Heras M., Ortin A., Cousens C., Minguijon E., Sharp J.M. (2003). Enzootic nasal adenocarcinoma of sheep and goats. Curr. Top. Microbiol. Immunol..

[115] Apostolidi E.D., Psalla D., Chassalevris T., Chaintoutis S.C., Giadinis N.D., Psychas V., Dovas C.I. (2019). Development of real-time PCR-based methods for the detection of enzootic nasal tumor virus 2 in goats. Arch. Virol..

[116] Cousens C., Minguijon E., Dalziel R.G., Ortin A., Garcia M., Park J., Gonzalez L., Sharp J.M., de las Heras M. (1999). Complete sequence of enzootic nasal tumor virus, a retrovirus associated with transmissible intranasal tumors of sheep. J. Virol..

[117] Ortin A., Cousens C., Minguijon E., Pascual Z., Villarreal M.P., Sharp J.M., Heras M.L. (2003). Characterization of enzootic nasal tumour virus of goats: Complete sequence and tissue distribution. J. Gen. Virol..

[118] De Las Heras M., Borobia M., Ortin A. (2021). Neoplasia-Associated Wasting Diseases with Economic Relevance in the Sheep Industry. Anim. Open Access J..

[119] Walsh S.R., Linnerth-Petrik N.M., Laporte A.N., Menzies P.I., Foster R.A., Wootton S.K. (2010). Full-length genome sequence analysis of enzootic nasal tumor virus reveals an unusually high degree of genetic stability. Virus Res..

[120] Walsh S.R., Stinson K.J., Menzies P.I., Wootton S.K. (2014). Development of an ante-mortem diagnostic test for enzootic nasal tumor virus and detection of neutralizing antibodies in host serum. J. Gen. Virol..

[121] Sid N., Belalmi N.E.H., Benhamza L., Ouhida S., Zebiri M.E., Aydogan A., Leroux C. (2018). First case report of enzootic nasal adenocarcinoma in "Ouled Djellal" ewe in Algeria. Open Vet. J..

[122] Lin X., Hao X., Zhao Z., Yu W., Gu Y., Bao G. (1995). Pathological study of goat nasal adenoma and adenocarcinoma. Acta Vcterinaria Et Zootechhnica Sin..

[123] He Y., Zhang Q., Wang J., Zhou M., Fu M., Xu X. (2017). Full-length genome sequence analysis of enzootic nasal tumor virus isolated from goats in China. Virol. J..

[124] Ye C., Huang Q., Chen T., Jiang J., Hou F., Xu D., Peng Y., Fang R., Chen J. (2019). First detection and genotypic analysis of goat enzootic nasal tumor virus 2 in Chongqing, China. Arch. Virol..

[125] Zhai S.L., Lv D.H., Xu Z.H., Yu J.S., Wen X.H., Zhang H., Chen Q.L., Jia C.L., Zhou X.R., Zhai Q. (2019). A Novel Enzootic Nasal Tumor Virus Circulating in Goats from Southern China. Viruses.

[126] Liu F., Feng Y.C., Yan Q.G., Han G.Q. (2011). Diagnosis of an enzootic nasal tumor of goat in Sichuan. Anim. Husb. Vet. Med..

[127] Jiang J.X., Lin Y.S., Jiang B., Mao K.M., You W., Zhang J.P., Hu Q.L. (2017). Molecular epidemiology of enzootic nasal tumor virus on goats in Fujian. Fujian J. Agric. Sci..

[128] Yu Y.D., Wei L.F., Huang X.J., Zhang B., Yu X.H., Tang C. (2014). Diagnosis of four cases of enzootic nasal adenocarcinoma of goats. Prog. Vet. Med..

[129] Hou H.Z., Zhang D., Hu X., Zhao R., Dai Y. (2018). Cloning and analysis of gag gene of enzootic nasal tumor virus in goats. China Herbiv. Sci..

[130] Xu G., Xian S., Zeng Z., Liang H., Wang B., Huang T., Tang D. (2018). Diagnosis of a goat intranasal tumor. China Herbiv. Sci..

[131] De las Heras M., Garcia de Jalon J.A., Minguijon E., Gray E.W., Dewar P., Sharp J.M. (1995). Experimental transmission of enzootic intranasal tumors of goats. Vet. Pathol..

[132] Walsh S.R., Stinson K.J., Wootton S.K. (2016). Seroconversion of sheep experimentally infected with enzootic nasal tumor virus. BMC Res. Notes.

[133] Wootton S.K., Metzger M.J., Hudkins K.L., Alpers C.E., York D., DeMartini J.C., Miller A.D. (2006). Lung cancer induced in mice by the envelope protein of jaagsiekte sheep retrovirus (JSRV) closely resembles lung cancer in sheep infected with JSRV. Retrovirology.

[134] Huang Q., Ye C., Chen T., Jiang J., Peng Y., Chen J., Fang R. (2019). EvaGreen-based real-time PCR assay for sensitive detection of enzootic nasal tumor virus 2. Mol. Cell. Probes.

[135] Hao H., Xie Z., Liao H., Liu D., Guo L., Liu J., Yang S., Shu L., Yan Q. (2014). Estabilshment of an RT-PCR method for detection of enzootic nasal tumor virus in goat. Chin. Vet. Sci..

[136] He R., Du Y., Gan L., Mohsin M.A., He B.X. (2021). Development of a SYBR Green-based real-time quantitative polymerase chain reaction assay to detect enzootic nasal tumor virus in goats. Can. J. Vet. Res. Rev. Can. De Rech. Vet..

[137] Zhang J.P., H J.J., Lin Y.S., You W., Liu D.Q., Mao K.M., Jiang B., Hu Q.L. (2021). A SYBR-Green I RT-qPCR assay for detecting enzootic nasal tumor virus in goats. Fujian J. Agric. Sci..

[138] Xiao S., Zhai S., Chen X., Xie Y., Zhou X., Lyv D., Wen X., Zhai Q., Jia C., Wei W. (2021). Development and Application of a Fluorescent PCR Method for Detection of Enzootic Nasal Tumor Virus. Chin. J. Anim. Infect. Dis..

[139] Davison A.J. (2010). Herpesvirus systematics. Vet. Microbiol..

[140] Thiry J., Saegerman C., Chartier C., Mercier P., Keuser V., Thiry E. (2008). Serological evidence of caprine herpesvirus 1 infection in Mediterranean France. Vet. Microbiol..

[141] Berrios P.E., McKercher D.G., Knight H.D. (1975). Pathogenicity of a caprine herpesvirus. Am. J. Vet. Res..

[142] Tempesta M., Camero M., Greco G., Pratelli A., Martella V., Buonavoglia C. (2001). A classical inactivated vaccine induces protection against caprine herpesvirus 1 infection in goats. Vaccine.

[143] Gonzalez J., Passantino G., Esnal A., Cuesta N., Garcia Vera J.A., Mechelli L., Saez A., Garcia Marin J.F., Tempesta M. (2017). Abortion in goats by Caprine alphaherpesvirus 1 in Spain. Reprod. Domest. Anim. Zuchthyg..

[144] Suavet F., Champion J.L., Bartolini L., Bernou M., Alzieu J.P., Brugidou R., Darnatigues S., Reynaud G., Perrin C., Adam G. (2016). First Description of Infection of Caprine Herpesvirus 1 (CpHV-1) in Goats in Mainland France. Pathogens.

[145] Tarigan S., Webb R.F., Kirkland D. (1987). Caprine herpesvirus from balanoposthitis. Aust. Vet. J..

[146] Chenier S., Montpetit C., Helie P. (2004). Caprine herpesvirus- 1 abortion storm in a goat herd in Quebec. Can. Vet. J. La Rev. Vet. Can..

[147] Camero M., Lanave G., Lucente M.S., Losurdo M., Di Paola G., Lorusso E., Martella V., Buonavoglia C., Tempesta M. (2019). Bubaline alphaherpesvirus 1 induces a latent/reactivable infection in goats. Comp. Immunol. Microbiol. Infect. Dis..

[148] Zell R., Delwart E., Gorbalenya A.E., Hovi T., King A.M.Q., Knowles N.J., Lindberg A.M., Pallansch M.A., Palmenberg A.C., Reuter G. (2017). ICTV Virus Taxonomy Profile: Picornaviridae. J. Gen. Virol..

[149] Anbalagan S., Hesse R.A., Hause B.M. (2014). First identification and characterization of porcine enterovirus G in the United States. PLoS ONE.

[150] Wang M., He J., Lu H., Liu Y., Deng Y., Zhu L., Guo C., Tu C., Wang X. (2017). A novel enterovirus species identified from severe diarrheal goats. PLoS ONE.

[151] Bunke J., Receveur K., Oeser A.C., Fickenscher H., Zell R., Krumbholz A. (2018). High genetic diversity of porcine enterovirus G in Schleswig-Holstein, Germany. Arch. Virol..

[152] Boros A., Pankovics P., Knowles N.J., Reuter G. (2012). Natural interspecies recombinant bovine/porcine enterovirus in sheep. J. Gen. Virol..

[153] Dong K., Hu J.Y., Li Z.C., Cai M.L., Zhang F., Wang W.Y., Wang Y.G., Wang X.P. (2021). Establishment and preliminary application of RT-PCR method for the detection of caprine/ovine enterovirus. Chin. J. Vet. Sci..

[154] Lu G. (2021). Epidemiological investigation and analysis of sheep enterovirus infection in some areas of Hebei province. Chin. J. Anim. Infect. Dis..

[155] Lu H.B., Hu J.Y., Zhang Q., Wang Y.G., Guo C.M., Zhu L.S., Tu C.C., Wang X.P. (2017). Development of a sandwich ELISA for detection of CEV antigen and epidemiological investigation. Chin. J. Vet. Sci..

[156] Wang R.D., Lin Q., Wang W.Y., Hu J.Y., Zhang Z.C., Zhu J.F., Wang X.P. (2020). Epidemiological investigarion and analysis of sheep enterovirus infection in Henan province. Chin. J. Vet. Sci..

[157] Li X., Hu J.Y., Lin Q., Wang X., Zheng B.W., Zhang Z.C., Hu L.P., Wang X.P. (2019). Epidemiological investigarion on enterovirus infection in cattle/sheep/goat herds in Shandong province. Chin. J. Vet. Sci..

